# Forecasting local COVID-19/Respiratory Disease mortality via national longitudinal shopping data: the case for integrating digital footprint data into early warning systems

**DOI:** 10.23889/ijpds.v8i3.2290

**Published:** 2023-09-11

**Authors:** James Goulding, Elizabeth Dolan, Gavin Long, Anya Skatova, John Harvey, Gavin Smith, Laila Tata

**Affiliations:** 1 N/LAB, University of Nottingham; 2 Bristol Medical School, University of Bristol; 3 Faculty of Medicine & Health Sciences, University of Nottingham

## Introduction & Background

The COVID-19 pandemic led to unparalleled pressure on healthcare services, highlighting the need for improved healthcare planning for respiratory disease outbreaks. With rapid virus diversification, and correspondingly rapid shifts in symptom expression, there is often a complete lack of representative clinical testing data available to modellers. This is especially true at the onset in outbreaks, where traditional epidemiological and statistical approaches that utilise case data ‘ground truths’ are extremely challenging to apply. In this abstract we preview the results of two novel studies that investigate how the use of digital footprint data - in the form of over-the-counter medication sales - might serve as a predictive proxy for underlying and often hidden disease incidence, and the extent to which such data might improve mortality rate forecasting at local area levels.

## Objectives & Approach

Over 2 billion transactions logged by a UK high-street health retailer were collated across English local authorities (n=314), generating weekly variables corresponding to a range of health purchase behaviours (e.g cough mixture / pain-relief sales) in each authority. These purchase data were additionally linked to a set of independent variables describing each local authority’s 1. weekly environment (e.g. weather, temperature, pollution), 2. socio-demographics (e.g. age distributions, deprivation levels, population densities) and 3. available local test case data. Machine learning regression models were then deployed to investigate the ability of each of these variable sets to underpin predictions of weekly registered deaths in the 314 authorities that were due to: COVID-19 between Apr 2020 - Dec 2021 (Study 1) or general respiratory disease between March 2016 - Mar 2020 (Study 2). All models were rigorously tested out-of-sample via walk forward cross-validation, and across a range of forecast windows.

## Relevance to Digital Footprints

Epidemics such as COVID-19 are recognised as being driven as much by behavioural factors as they are by clinical ones. Indicators of infection rates may be revealed in purchasing and self-medication logs, where there exists rich data: in 2022 UK citizens were reported to generate >1 billion prescriptions; consume ~6,300 tonnes of paracetamol; and spend £572m on cough, cold and sore throat treatments. Application of the digital footprint data logs generated by such activities may hold potential to reveal hidden disease incidence and risk to vulnerable communities, without reliance on prohibitively expensive testing infrastructures.

## Results

Evidence was found that models incorporating digital footprint sales data were able to significantly out-perform models that used variables traditionally associated with respiratory disease alone (e.g. sociodemographics, weather, or case data). In Study 1, XGBoost models were able to optimally predict the number of COVID deaths 21 days in advance (R2=0.71***), significantly outperforming models based on official COVID case data alone at local-area levels (R2=0.44**). For the pre-COVID period, where registered deaths express a far greater seasonal pattern, models optimally predicted registered respiratory deaths 17 days in advance (R2=0.78***), with highest accuracy gains over models without digital footprint data (increases in R2 between 0.09 to 0.11) occurring in periods of maximum risk to the general public (winter periods).

## Conclusions & Implications

Over-the-counter medication purchases related to management of respiratory illness are correlated with registered deaths at a 17-21 day window. Results demonstrate the potential for sales data to support early warning population health mechanisms at local area levels, and the need for ongoing research into their application to support health planning.

